# *ABCA4* mutations in Portuguese Stargardt patients: identification of new mutations and their phenotypic analysis

**Published:** 2009-03-25

**Authors:** Susana Maia-Lopes, Jana Aguirre-Lamban, Miguel Castelo-Branco, Rosa Riveiro-Alvarez, Carmen Ayuso, Eduardo Duarte Silva

**Affiliations:** 1 Visual Neuroscience Laboratory, IBILI, Faculty of Medicine, Coimbra, Portugal; 2 Genetics Department, Fundacíon Jiménez Díaz and CIBER de Enfermedades Raras (CIBERER), Madrid, Spain; 3 Centre for Hereditary Eye Diseases, Department of Ophthalmology, University Hospital Coimbra, Coimbra, Portugal

## Abstract

**Purpose:**

To resolve the spectrum of causative retina-specific ATP-binding cassette transporter gene (*ABCA4*) gene mutations in Portuguese Stargardt (STGD) patients and compare allele frequencies obtained in this cohort with those of previous population surveys.

**Methods:**

Using a microarray technique (ABCR400 gene chip), we screened all previously reported *ABCA4* gene mutations in the genomic DNA of 27 patients from 21 unrelated Stargardt families whose phenotypes had been clinically evaluated using psychophysics and electrophysiological measurements. Furthermore, we performed denaturing high performance liquid chromatography whenever one or both mutant alleles failed to be detected using the *ABCR* gene chip.

**Results:**

A total of 36 mutant alleles (out of the 54 tested) were identified in STGD patients, resulting in a detection rate of 67%. Two mutant alleles were present in 12 out of 21 STGD families (57%), whereas in four out of 21 (19%) of the families, only one mutant allele was found. We report the presence of 22 putative pathogenic alterations, including two sequence changes not found in other populations, c.2T>C (p.Met1Thr) and c.4036_4037delAC (p.Thr1346fs), and two novel disease-associated variants, c.400C>T (p.Gln134X) and c.4720G>T (p.Glu1574X). The great majority of the mutations were missense (72.7%). Seven frameshift variants (19.4%), three nonsense mutations (8.3%), and one splicing sequence change (2.7%) were also found in STGD chromosomes. The most prevalent pathologic variant was the missense mutation p.Leu11Pro. Present in 19% of the families, this mutation represents a quite high prevalence in comparison to other European populations. In addition, 23 polymorphisms were also identified, including four novel intronic sequence variants.

**Conclusions:**

To our knowledge, this study represents the first report of *ABCA4* mutations in Portuguese STGD patients and provides further evidence of different mutation frequency across populations. Phenotypic characterization of novel putative mutations was addressed.

## Introduction

Stargardt disease (STGD) is an autosomal recessive macular dystrophy characterized by a childhood or juvenile onset. STGD accounts for 7% of all retinal dystrophies and affects about 1 in 10,000 individuals [1,2].

Kaplan et al. [2,3] mapped the Stargardt/fundus flavimaculatus disease (STGD/FFM; OMIM 248200) to chromosome 1p21-p22. Molecular analysis of the ATP-binding cassette transporter gene (*ABCA4*) gene, performed by several groups, led to the identification of more than 490 sequence variations [4-7]. However, such high allelic heterogeneity within the 50 exons of *ABCA4* gene makes it difficult to predict the disease-causing variants. It is likely that the wide variation in retinal phenotypes may be explained by different combinations of *ABCA4* mutations. Therefore, the severity of phenotype is partly conditioned by the severity of mutant allele(s) [8,9]. Furthermore, mutations in this gene have also been implicated in other retinal dystrophies, namely autosomal recessive cone-rod dystrophy (arCRD; OMIM 604116), retinitis pigmentosa (OMIM 601718), and to an increased predisposition to age-related macular degeneration (AMD; OMIM 153800) [4,10-12]. The detection of so many sequence variants has enabled a heterogeneous frequency of disease-associated alleles to be reported across populations [6,9,12-18].

*ABCA4* encodes a retina-specific ATP-binding cassette (ABC) transporter protein that resides at the rim of cones and rods outer segment discs and is involved in the all-trans-retinal transport generated by activation of opsins [19]. An important pathological feature of STGD/FFM and AMD is the abnormal accumulation of lipofuscin in the retinal pigment epithelium cells (lipofuscin accumulation occurs also in normal aging). Progressive atrophy of retinal pigment epithelium and degeneration of the underlying photoreceptors are thought to be the cause of bilateral loss of central vision.

Here, we report the Portuguese population-specific *ABCA4* mutant alleles found in a cohort of STGD patients. Our goal was to further contribute to the establishment of genotype-phenotype correlations involving *ABCA4*.

## Methods

### Patients

Molecular screening of *ABCA4* was performed in 27 STGD patients from 21 Portuguese families. Patients and their family members were clinically evaluated at the University Hospital of Coimbra (Coimbra, Portugal). Clinical diagnosis of STGD was based on full ophthalmologic examination, including assessment of best-corrected visual acuity, slit-lamp examination, dilated fundus photography, fluorescein angiography, color vision testing, full-field electroretinograms (ERG) and multifocal ERGs (mfERGs) [20]. The criteria for STGD phenotype included bilateral central vision loss and pigmentary macular lesions, normal caliber of retinal vessels, absence of pigmented bone spicules, and compatibility with recessive mode of inheritance. We staged our patients according to the severity criteria of central fundus changes described previously by Scholl and colleagues [21]. After the objectives of the study were explained to each participant, informed consent was obtained, and a peripheral blood sample was collected and preserved frozen. The research was conducted in accordance with the tenets of the Declaration of Helsinki and with the institutional guidelines defined by the ethics committee of the Faculty of Medicine of Coimbra.

### Mutation analysis of *ABCA4*

Genomic DNA was extracted using an automated DNA extractor (BioRobot EZ1, Qiagen, Hilden, Germany). All the exons of *ABCA4* (GDB370748, GenBank U88667.1) were PCR-amplified as described previously [15] and used in the primer extension reaction (APEX) on the ABCR400 microarray, which is, in essence, a sequencing reaction on a solid support, as described elsewhere in the literature [7]. In short, 5′-modified sequence specific oligonucleotides are arrayed on a glass slide. In general, these oligonucleotides are designed with their 3′ end immediately adjacent to the variable site. PCR-prepared and fragmented target nucleic acids are annealed to oligonucleotides on the slide, followed by sequence-specific extension of the 3′ ends of primers with dye-labeled nucleotide analogs (ddNTPs) by DNA polymerase. Additionally, amplified fragments were subjected to denaturing high performance liquid chromatography (dHPLC) screening whenever any or both mutant alleles had failed to be identified in STGD patients, using a WAVE^TM^ DNA Fragment Analysis System (Transgenomic, San Jose, CA), in which the temperature conditions of dHPLC were designed and validated for all 50 exons, as described by other [22]. All abnormal heteroduplexes obtained were then sequenced. Amplification products were purified with QIA-quick Gel Extraction Kit (Qiagen). Sequencing reactions were performed using the four-dye terminator cycle sequencing ready reaction kit (BigDye DNA Sequencing Kit; Applied Biosystems, Foster City, CA). Sequencing products were purified through fine columns (Sephadex G-501; Princetown Separations, Adelphia, NJ) and resolved in an ABI Prism 3130 (Applied Biosystems).

Analysis of haplotypes was performed in those families whose patients had more than one mutation for the following three microsatellite markers flanking the *ABCA4* gene: TEL-D1S435 (89.81 Mb), D1S2804 (91.13 Mb), and *ABCA4*-D1S236 (93.06 Mb)-CEN. Samples were analyzed in an automatic genetic analyzer (ABIprism 3130, Applied Biosystems).

Control samples were selected from 55 unrelated healthy individuals who did not have a personal or familiar history of retinal disease. Anonymous blood donors were recruited at the University Hospital of Coimbra.

## Results

In all, 27 patients from 21 Portuguese STGD families were evaluated. Our study detected 18 previously reported mutations and 4 sequence change unreported in other populations, including 2 novel disease-associated variants (Table 1). A total of 36 mutant alleles (out of the 54 tested) were identified in STGD patients. Gene chip screening allowed us to achieve a mutation detection rate of 55%. When we consider the combination of microarray and dHPLC technologies, we were able to identify two mutant alleles in 12 out of 21 STGD families (57%), whereas we found only one mutant allele in four out of 21 (19%) of the families. No mutation was detected in the remaining five families (24%). Therefore, with the combined strategy a detection rate of 67% was obtained. Most disease alleles carried missense mutations (27/36 corresponding to 72.7%). However, frameshift variants (7/36; 19.4%), nonsense mutations (3/36; 8.3%), and one splicing sequence change (2.7%) were also identified in STGD chromosomes. When patients had more than one mutation, allelic segregation analyses of the families (including parents and siblings) was performed to establish the haplotype and disease-associated haplotypes cosegregated within all families analyzed. Interestingly, four disease alleles were found to be double mutants (families 5, 11, and 19), two of them carried by one STGD patient. These findings led to the identification of the following three variants acting in *cis* (complex alleles): p.[Ser1642Arg]+[Val1681_Cys1685del], found in 9.5% of the families; p.[Val931Met]+[Ser1642Arg], found in 4.8% of the families, and p.[Met1Val]+[Arg2030Gln], found in 4.8% of the families (for details, see Table 1). Most of the mutations detected have been reported as STGD-associated variants: p.Met1Val, p.Asn96Asp, p.Arg290Trp, p.Val931Met, p.Gly1961Glu, p.Leu2027Phe, p.Arg2030Gln, p.Asp1048fs, and IVS40+5G>A.

**Table 1:** ABCA4 gene (GDB370748, GenBank U88667.1) mutations identified in Portuguese STGD patients.

**Family**	**Patient**	**CFC**	**Onset (age)**	**VA (OD/OS)**	**Nucleotide changes (exons)**	**Effect changes [references]**
1	4427	S	7	1/10 / 1/10	c.286A>G(3) / c.4139C>T(28)	p.Asn96Asp [30]/p.Pro1380Leu [13]
	4413	S	14	1/10 / 0.5/10	c.286A>G(3) / c.4139C>T(28)	p.Asn96Asp [30]/p.Pro1380Leu [13]
	4454	S	11	1/10 / 1/10	c.286A>G(3) / c.4139C>T(28)	p.Asn96Asp [30]/p.Pro1380Leu [13]
2	4458	Mi	5	8/10 / 6/10	ND / ND	ND/ND
	4455	S	8	1/10 / 8/10	ND / ND	ND/ND
3	4431	Mo	6	1,6/10 / 1,6/10	c.1804C>T(13) / c.IVS+5G>A(40)	p.Arg602Trp [30]/SPLICE [11]
4	4626	S	6	FC / FC	c.3211_3212insGT(22) / c.3211_3212insGT(22)	p.Asp1048fs [5]/p.Asp1048fs [5]
5	4514	S	12	1/10 / 1/10	c.32T>C(1) / c.[1A>G(1)]+[6089G>A(44)]	p.Leu11Pro [12]/p.(Met1Val [6])+(Arg2030Gln [9])
6	4525	Mo	14	1/10 / 1/10	ND / c.868C>T(8)	ND/p.Arg290Trp [6]
7	4585	Mo	11	0.5/10 / 0.5/10	c.6079C>T(44) / ND	p.Leu2027Phe [5]/ND
8	4678	Mo	9	0.5/10 / 1/10	c.3113C>T(21) / c.3602T>G(24)	p.Ala1038Val [5]/p.Leu1201Arg [9]
9	4675	Mo	7	0.5/10 / 1/10	c.2T<C(1) / c.2T<C(1)	p.Met1Thr/p.Met1Thr
10	4737	Mo	24	1.2/10 / 1.2/10	c.5882G>A(42) / c.3211_3212insGT(22)	p.Gly1961Glu [4]/p.Asp1048fs
11	4613	S	9	FC / FC	c.[4926C>G(35)]+[5041_5055del(36)] / c.32T>C(1)	p.(Ser1642Arg [10])+(Val1681_Cys1685del [10])/p.Leu11Pro
12	4796	Mo	43	1/10 / 3/10	c.4720G>T(33) / c.2791G>A(19)	p.Glu1574X/p.Val931Met [5]
13	4859	Mo	30	0.5/10 / 0.5/10	c.5882G>A(42) / ND	p.Gly1961Glu/ND
	5472	Mo	30	6/10 / 8/10	c.5882G>A(42) / ND	p.Gly1961Glu/ND
14	4974	S	7	1/10 / 1/10	c.4036_4037delAC(27) / c.400C>T(4)	p.Thr1346fs/p.Gln134X
	4975	S	7	1/10 / 1/10	c.4036_4037delAC(27) / c.400C>T(4)	p.Thr1346fs/p.Gln134X
15	5193	Mo	9	1/10 / 1/10	ND / ND	ND/ND
16	5138	Mo	27	1/10 / 1/10	ND / ND	ND/ND
17	5111	Mo	29	2.5/10 / 1.6/10	c.1928T>G(13) / ND	p.Val643Gly/ND
	5137	Mo	25	3/10 / FC	ND / c.32T>C(1)	ND/p.Leu11Pro
18	5709	Mi	9	2/10 / 2/10	c.32T>C(1) / c.1804C<T(13)	p.Leu11Pro/p.Arg602Thr [9]
19	5434	Mo	17	2/10 / 2/10	c.[2791G>A(19)]+[4926C>G(35)] / c.[4926C>G(35)]+[5041_5055del(36)]	p.[Val931Met]+[Ser1642Arg]/ p.[Ser1642Arg]+[Val1681_Cys1685del]
20	5689	Mi	1	1/10 / 1/10	ND / ND	ND/ND
21	5917	Mi	9	0.5/10 / 2/10	ND / ND	ND/ND

The translation start codon ATG/methionine is numbered +1. Two sequence changes unreported in other populations [c.2T>C (p.Met1Thr) and c.4036_4037delAC (p.Thr1346fs)] and two novel disease-associated variants [c.400C>T (p.Gln134X) and c.4720G>T (p.Glu1574X)] were found (Families 9, 12 and 14). Exons affected and references of previously reported mutations are indicated in the columns ‘nucleotide changes’ and 'effect changes', respectively. CFC states for central fundus changes and is a measure of disease severity (Mi-mild; Mo-moderate; S-severe), as described previously by others [21].

Although most of the mutations were found in one family, five disease-associated alleles were detected in unrelated STGD families (p.Leu11Pro, p.Asp1048fs; p.Gly1961Glu; p.Ser1642Arg; p.Val1681_Cys1685del; p.Val931Met). The most prevalent disease-associated variant was the missense mutation p.Leu11Pro, accounting for 11% (4/36) of the disease chromosomes. This variant was present in four out of 21 families (19%). The p.Gly1961Glu mutation, associated with AMD, was found in 9.5% of our patients. This is a common variant observed in patients of European origin,.

**Figure 1:**
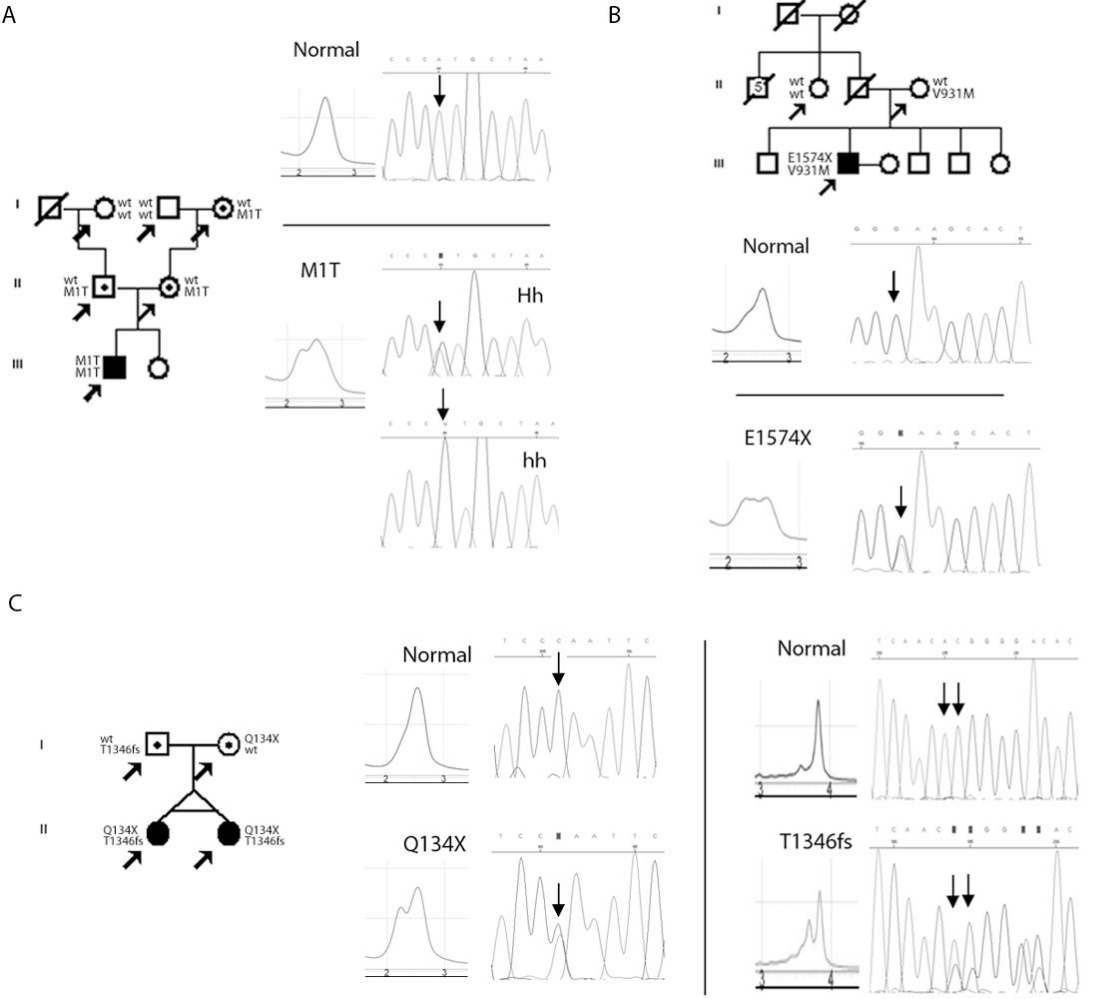
Pedigrees, elution dHPLC profiles, and sequence changes for each new disease-associated ABCA4 mutations are shown: **A** - p.Met1Thr (c.2T>C); **B** - p.Glu1574X (c.4720G>T); **C** - p.Gln134X (c.400C>T); and p.Thr1346fs (c.4036_4037delAC). In **B** and **C**, the forward sequence changes are shown. In **A**, the reverse normal, heterozygous, and homozygous mutant sequences are presented. The arrows indicate the individuals genotyped from each family, including the STGD proband (filled symbols) and siblings.

Among the four null mutations unreported in other populations, the c.2T>C transition at the initiation codon (p.Met1Thr) was found in homozygous state in only one STGD patient (Family 9; Figure 1A). This double null mutant STGD patient had an early onset and showed moderate macular changes and a dramatic visual loss two years after disease onset. Familial segregation was confirmed, and consanguinity was denied by the family. However, both branches of the family come from neighboring villages. This missense mutation p.Met1Thr was absent in the 102 chromosomes from healthy unrelated Portuguese controls and was recently reported by us in a STGD relative [20].

The STGD patient from Family 12 is a compound heterozygous with p.Val931Met and a novel nonsense mutation at exon 33 (p.Glu1574X; Figure 1B). Disease onset for this patient was at age 43. Ophthalmic examination revealed moderate central retinal changes, decreased mfERG responses exclusively in the central 15 degrees, and decreased visual acuity.

Finally, two null mutations were identified in a Brazilian family of Portuguese ancestry (Family 14; Figure 1C). Both severely affected patients (monozygous twins) were found to be compound heterozygous with a novel nonsense mutation at exon 4 (p.Gln134X) and a frameshift in exon 27 caused by a deletion (c.4036_4037delAC leading to p.Thr1346fs); see Figure 1C. This last mutation was recently found by us in a STGD relative [20], but has not been reported in other populations.

Several polymorphisms were also identified and are summarized in Table 2. In all, 23 polymorphic changes were detected, four of which are novel intronic putative nonpathogenic variants (IVS7+8T>C; IVS14+47T>C; IVS19+34C>T; IVS22–19G>A). Allelic segregation analyses were performed in all families whose patients had more than one mutation (except Family 19). Disease-associated haplotypes were found to be segregated within the families.

**Table 2:** *ABCA4* polymorphisms (GDB370748, GenBank U88667.1) detected in Portuguese STGD patients.

**Exon**	**Nucleotide Change**	**Effect**	**STGD Families**	**Frequency**	**References**
IVS3	c.302+20C>T	-	12	4.8%	[6]
IVS3	c.302+26A>G	-	7,12,13,14	1.91%	[6]
6	c.635G>A	p.Arg212His	13,19	9.5%	[15]
IVS7	c.859+8T>C	-	17	4.8%	Present study
10	c.1268A>G	p.His423Arg	2,4,5,6,10,11,12,13,14,18,19	53%	[13]
10	c.1269C>T	p.His423His	16	4.8%	[13]
IVS10	c.1356+5delG	SPLICE	1,7,11,15,20	23.8%	[13]
IVS14	c.2161+47T>C	−	18	4.8%	Present study
19	c.2828G>A	p.Arg943Gln	3,10,18,19	19.1%	[5]
IVS19	c.2919+34C>T	-	12	4.8%	Present study
20	c.2964T>C	p.Leu988Leu	12	4.8%	[6]
IVS22	c.3326–19G>A	-	2	4.8%	Present study
IVS33	c.4773+48C>T	Splice	1,2,3,5,6,8,9,10,12,13,14,16,17,18,19,20	76.2%	[13]
40	c.5603A>T	p.Asn1868Ile	4,10,17	14.3%	[6]
40	c.5682G>C	p.Leu1894Leu	1,2,4,5,8,10,12,13,17,18	47.6%	[6]
41	c.5814A>G	p.Leu1938Leu	1,2,5,8,10,12,13,18	3.81%	[6]
42	c.5843CA>TG/c.5843C>T	p.Pro1948Leu	11	4.8%	[14]
42	c.5844A>G	p.Pro1948Pro	1,2,5,8,10,12,13	33.3%	[14]
44	c.6069C>T	p.Ile2023Ile	9,12,14,19	19.1%	[6]
45	c.6249C>T	p.Ile2083Ile	9,12,14,19	19.1%	[5]
46	c.6285T>C	p.Asp2095Asp	1,2,8,9,10,12,14,19	38.1%	[14]
IVS48	c.6769+21C>T	SPLICE	1,10	9.5%	[6]
49	c.6764G>T	p.Ser2255Ile	1,9,14,19	19.1%	[5]

Several polymorphisms in exons and introns (IVS) throughout the entire *ABCA4* gene were found in our study population. Four of those polymorphisms were novel, being first described in the present study. References of polymorphisms previously reported are indicated. The ‘A’ of the start codon ATG/methionine is numbered +1.

## Discussion

To our knowledge, this is the first report of *ABCA4* mutations in Portuguese STGD patients. To date, over 490 variants have been reported in the largest gene of the ABC family: *ABCA4* gene. Some of the variants that have been described are rare and may even be specific to certain specific geographic areas. Therefore different frequency distribution across populations have been reported. In this cohort of Portuguese patients, the most prevalent variant found in 19% of the families, Leu11Pro, is considered a rare mutation in other populations. Even in Spain, and in spite of its geographic proximity, Leu11Pro frequency is significantly lower (<1%) in macular degenerations associated with *ABCA4* mutations [17,23]. This likely moderate missense substitution, involving a conserved nonpolar amino acid residue, is located in the intracytoplasmic domain of the ABCA4 protein; it has been reported in FFM and in arCRD [12,23]. The most frequent mutation in various European countries is p.Gly1961Glu. Although its frequencies range between 11%–21%, it seems to be less common in the Portuguese population (9.5%). Its prevalence is similar to the one found in other south European populations, namely 6.6% in Spanish STGD patients [7,23]. Interestingly, the p.Arg1129Leu, which is the most frequent variant in Spain (14.5%) [20], was not found in any of the 21 STGD Portuguese families studied. These findings are consistent with studies on genetic diversity. Those studies concluded that the Iberian population is not a genetic edge of European variation and might have a higher level of diversity than some neighboring populations, receiving significant North and sub-Saharian African influences at different times [24]. Therefore, this study might provide further evidence of the importance of molecular analysis of this considerable large and polymorphic gene in different populations.

Also identified in our patient population were four putative pathogenic mutations, two of which are novel variants, that have not been reported in other populations: Family 9, c.2T>C (p.Met1Thr); Family 14, c.400C>T (p.Gln134X) and c.4036_4037delAC (p.Thr1346fs); and Family 12, c.4720G>T (p.Glu1574X). The p.Met1Thr substitution was detected in both chromosomes of a severely affected STGD patient, whose heterozygous mother presented subclinical impairment of retinal function even in absence of any fundus change, as described in our previous report [20]. Interestingly, this variant is the second substitution residing in the Met1 residue. Previously, Briggs and colleagues [6] described the p.Met1Val in a heterozygous STGD patient with no other sequence change identified. Therefore, this null mutation may lead to early disease onset, moderate central fundus changes (even as early as one year after disease onset), and residual visual acuity when in the homozygous state. Future functional studies should assess the relative severity of this variant to clarify whether this mutation can be pathologic even in heterozygous state as has been suggested for other *ABCA4* mutations, namely p.Gly1961Glu [25-27]. The novel nonsense mutation, a G>T transversion leading to p.Glu1574X, involves a highly conserved nucleotide in the ortholog bovine and mouse proteins. This sequence change causes a protein truncation before NBD-2, a functional domain that is believed to diminish ATP hydrolysis by NBD-1, without altering the basal ATPase activity [28]. Segregation analysis was limited to available relatives: the mother, from whom the p.Val931Met al.lele was inherited, and the paternal aunt, who did not carry any of these disease alleles. The p.Val931Met mutation resides in the NBD-1, what according to the model proposed by Sun et al., has a severe impact in ABCA4 protein function, eliminating both basal and retinal-stimulated ATPase activity [28]. Interestingly, clinical examination of this patient revealed that retinal damage was limited to the central 15 degrees, a relative late onset and moderate central fundus changes after the second year of disease onset. Therefore, the combination of the two alleles results in a relatively late disease onset with a moderate retinal dysfunction progression.

In Family 14, compound heterozygous of a nonsense mutation at exon 4 (p.Gln134X) and a frameshift in exon 27 (p.Thr1346fs), were associated to a very early disease onset. Even after only 4 years of disease onset, both patients were severely affected and shared dramatically reduced visual acuities. Their mfERG results revealed severely decreased response amplitudes (almost abolished) within the central 30 degrees of the retina and impaired color vision in all three main chromatic axes. This stop mutation affects a 100% conserved nucleotide, before any of the ATP binding domains, leading to a premature stop codon of 134 amino acid residue out of the 2,273 residues of ABCA4 protein that likely undergo nonsense-mediated decay. Additionally, in this family, we found a second null mutation that resides between the two homologous halves of ABCA4, a deletion of a dinucleotide AC at codon 1346. An insertion of a dinucleotide CA affecting the same codon has been detected and has been found to be associated with a severe phenotype (arCRD) [6]. Therefore, both novel variants are compatible with the dramatically severe phenotype observed in these STGD patients.

Interestingly, in patients from families 9 and 14, two null mutant alleles were identified in each patient. It is worth noting that even considering the severe phenotype presented by those patients, they were diagnosed with STGD disease. However, according to the proposed model, the combination of two null alleles likely accounts to a more severe phenotype as retinitis pigmentosa or arCRD [29]. Since in both families, STGD patients had short disease progression, we speculate whether their phenotype may evolve (in later stages) to a more severe retinal impairment such as arCRD.

In this study, genotype-phenotype correlations were addressed based on previous extensive phenotype characterization. However, the value of the ABCA4 model proposed for genotype-phenotype might be limited in larger families because of the intrafamiliar phenotypic variation and since the model is mainly based on large set of single patients, not on extensive families. Mutation analysis was performed with a combination of complementary techniques: *ABCA4* gene chip, dHPLC, and direct sequencing. This was found to be a successful strategy resulting in high mutation rate detection (67%), compared to other surveys [6,18,30,31]. It is worth to note the efficiency, specificity and high detection rate of the ABCR400 chip. However, in 3 out of the 21 studied families (14.3%; families 9, 12, and 14), the causal mutations were detected using a combination of dHPLC and direct sequencing technology.

Functional studies to evaluate the biochemical defects caused by the numerous variants identified will help understanding the relative impact of the complex (and single) heterozygous. Therefore, those studies will improve genetic counselling of families affected with ABCA4-related retinal diseases.
